# Enhancement of neurogenesis and cognition through intranasal co-delivery of galanin receptor 2 (GALR2) and neuropeptide Y receptor 1 (NPY1R) agonists: a potential pharmacological strategy for cognitive dysfunctions

**DOI:** 10.1186/s12993-024-00230-5

**Published:** 2024-03-28

**Authors:** Raquel Sánchez-Varo, Alexander López-Salas, Rasiel Beltran-Casanueva, Estela Díaz-Sánchez, Jose Erik Alvarez-Contino, Miguel Angel Barbancho-Fernández, Pedro Serrano-Castro, Kjell Fuxe, Dasiel O. Borroto-Escuela, Natalia García-Casares, Manuel Narváez

**Affiliations:** 1 NeuronLab. Departamento Fisiología Humana, Histología Humana, Anatomía Patológica y Educación Física y Deportiva, Facultad de Medicina, Universidad de Malaga, 29071 Malaga, Spain; 2 Instituto de Investigación Biomédica de Málaga-IBIMA-Plataforma Bionand, Universidad de Malaga, 29071 Malaga, Spain; 3 Centro de Investigación Biomédica en Red Sobre Enfermedades Neurodegenerativas (CIBERNED), 28031 Madrid, Spain; 4 Department of Neuroscience, Karolinska Institutet, Stockholm, Sweden; 5 Receptomics and Brain Disorders Lab, Universidad de Málaga. Facultad de Medicina., Edificio Lopez-Peñalver, Jimenez Fraud 10, 29071 Málaga, Spain; 6 Vithas Málaga., Vithas Málaga. Grupo Hospitalario Vithas, Málaga, Spain; 7 Instituto de Investigación Biomédica de Málaga, Unit of Neurology, Hospital Regional Universitario de Málaga, Málaga, Spain; 8 Departamento de Medicina y Dermatología. , Facultad de Medicina. Universidad de Málaga. , Málaga, Spain

**Keywords:** Neurogenic enhancement, Cognitive enhancement, Intranasal administration, GALR2 agonists, NPY1R agonists, M1145, Spatial memory performance, Neuronal survival, Neuronal differentiation

## Abstract

**Background:**

Spatial memory deficits and reduced neuronal survival contribute to cognitive decline seen in the aging process. Current treatments are limited, emphasizing the need for innovative therapeutic strategies. This research explored the combined effects of intranasally co-administered galanin receptor 2 (GALR2) and neuropeptide Y1 receptor (NPY1R) agonists, recognized for their neural benefits, on spatial memory, neuronal survival, and differentiation in adult rats.

After intranasal co-delivery of the GALR2 agonist M1145 and a NPY1R agonist to adult rats, spatial memory was tested with the object-in-place task 3 weeks later. We examined neuronal survival and differentiation by assessing BrdU-IR profiles and doublecortin (DCX) labeled cells, respectively. We also used the GALR2 antagonist M871 to confirm GALR2's crucial role in promoting cell growth.

**Results:**

Co-administration improved spatial memory and increased the survival rate of mature neurons. The positive effect of GALR2 in cell proliferation was confirmed by the nullifying effects of its antagonist. The treatment boosted DCX-labeled newborn neurons and altered dendritic morphology, increasing cells with mature dendrites.

**Conclusions:**

Our results show that intranasal co-delivery of GALR2 and NPY1R agonists improves spatial memory, boosts neuronal survival, and influences neuronal differentiation in adult rats. The significant role of GALR2 is emphasized, suggesting new potential therapeutic strategies for cognitive decline.

**Supplementary Information:**

The online version contains supplementary material available at 10.1186/s12993-024-00230-5.

## Background

The established belief that the process of human adult neurogenesis is a lifelong phenomenon has captured significant scientific attention, especially considering its critical role in various neurological conditions, including Alzheimer's disease, Huntington's disease, Parkinson’s disease, dementia with Lewy bodies, frontotemporal dementia, and others [1–5]. The pressing nature of Alzheimer's disease, which constitutes approximately 70% of all dementia cases worldwide, impacting nearly 35 million individuals, underscores the urgency to devise novel therapeutic strategies. Enhancing hippocampal neurogenesis emerges as a promising approach, particularly in light of exacerbated cognitive and psychiatric symptoms in Alzheimer's patients following COVID-19 infection [6, 7].

Within the dentate gyrus of the hippocampus, a considerable amount of neurons are produced, yet only a select few survive for long-term integration into the network, a process governed by activity-dependent regulation [8]. It is during this stage of development that cells begin to express doublecortin (DCX), a protein critical for neuronal differentiation, with significant roles in cell migration and potentially other maturational aspects, including synaptogenesis [9, 10]. Uniquely expressed within neurogenesis-contributing cells in the dentate gyrus, DCX marks a crucial phase of neural development, indicated by neurite elongation [10, 11].

Recent research, notably by Canatelli-Mallat et al. [12], has highlighted that in middle-aged (MA) rats, a decrease in immature (DCX-positive) neurons in the dorsal hippocampus does not correlate with performance in object recognition tasks, yet shows significance in spatial memory tasks. This emphasizes the specific role of the dorsal hippocampus in spatial context and object recognition, whereas other brain regions, such as the prefrontal cortex, are instrumental in recognizing object features [13].

It is worth noting, however, that mature granule cells lack DCX expression. The transient phase of DCX expression, observable in the growth cones of dendrites and axons, typically persists for an average of three weeks [14, 15]. Significantly, the period of DCX expression overlaps with neuronal migration, a pivotal process in neurodevelopment [16]. Intriguingly, the majority of these newly generated cells remain primarily within the inner third of the granule cell layer, suggesting a specific constraint or specificity in DCX-guided migration. DCX's cytoplasmic localization in immature neurons provides a powerful tool for studying morphology through immunohistochemical methods [17–19]. This evidence underlines the central role of DCX in the sophisticated processes of neuronal development, with implications for understanding neurogenesis, neuronal migration, and maturation.

Emerging research has spotlighted neuropeptides, specifically Neuropeptide Y (NPY) and Galanin (GAL), as pivotal controllers of these neurogenic niche activities. NPY, its receptors, particularly the NPY Y1 receptors (NPY1R), are implicated in essential biological and pathophysiological functions, including mood regulation, neuronal excitability, neuroplasticity, and memory [20, 21]. Previous studies have showcased NPY's crucial role in fostering neurogenesis within hippocampal stem cells, both in vitro and in vivo [22, 23]. Interestingly, enhanced hippocampal NPY mRNA expression has been observed in rats following spatial learning tasks [24]. However, aging rats have shown a correlation between declining NPY expression in the hippocampus and deterioration in memory and neurogenesis [25]. Reduced NPY receptor densities and NPY levels in cerebrospinal fluid and plasma have been observed in Alzheimer's disease patients, making NPY1R attractive targets for enhancing dentate neurogenesis and spatial learning [26–29].

Like NPY, Galanin (GAL) is widespread in the central nervous system and has diverse physiological impacts [30]. GAL's effects on hippocampal neurogenesis are only recently understood, with the GAL 2/3 receptor agonist, GAL 2–11, shown to foster proliferation and trophism in progenitor cells [31]. GAL's influence on memory is dose- and site-dependent, with outcomes ranging from enhancing learning to exhibiting no effect or even inhibitory impacts [32]. Moreover, GALR2 receptors have shown to mediate memory-enhancing and hippocampal toxicity-inhibiting effects in an Alzheimer's disease rat model [33].

Previous research efforts have underscored the interaction between Neuropeptide Y (NPY) and Galanin (GAL) via specific NPY1R-GALR2 heteroreceptor complexes in interconnected brain regions, like the amygdala, dorsal and ventral hippocampus, and various hypothalamic regions. These interactions have profound implications for the neurological disorders mentioned earlier [34–39]. In particular, we explored whether NPY1R and GALR2 agonists stimulate proliferation in DG neuronal precursors in vivo at 24 h [37, 39].

For this study, we infused both peptides into rats using a novel intranasal delivery method. The intranasal route of drug administration has garnered significant attention in neuroscientific research due to its potential to deliver therapeutic agents directly to the central nervous system, bypassing the blood–brain barrier (BBB). This method offers a non-invasive approach, reducing systemic side effects, ensures rapid absorption and onset of action, making it particularly advantageous for conditions requiring immediate therapeutic intervention. Given these benefits, our study's choice of intranasal administration aligns with the growing trend in neuroscience to explore efficient and patient-friendly drug delivery methods [40]. We combined this with BrdU labeling and subsequently studied the phenotype of the newly generated cells in the dentate gyrus 3 weeks following the treatment. This period was chosen based on prior research that indicates new granule neurons can contribute functionally and behaviorally to hippocampal function as early as 2–3 weeks of age [41, 42]. However, other studies suggest that these new neurons might not substantially contribute to behavior until they reach about 6–8 weeks of age [43, 44]. It is essential to recognize that different species contribute to these diverging results. Studies indicating earlier functionality of new neurons were conducted in rats, while those suggesting more delayed function were performed in mice. This inconsistency might suggest that new neurons mature more rapidly or contribute more significantly to hippocampal function in rats than in mice [45].

In conclusion, we place a heavy emphasis on the importance of adult neurogenesis under physiological conditions. Our study brings to light the potential regulatory role of neuropeptides, specifically NPY and GAL, in these processes, offering promising therapeutic avenues for addressing age-related cognitive decline and the potential early stages of cognitive impairment. By further exploring the implications of NPY1R and GALR2 agonists on neurogenesis and cognition, our research aims to build a more comprehensive understanding of these intricate biological phenomena.

## Materials and methods

### Animals

Male Sprague–Dawley rats, 6–8 weeks old and weighing between 200-250 g, were obtained from CRIFFA (Barcelona). The rats had free access to food and water and were maintained under a standard 12 h dark/light cycle, with controlled relative humidity (55–60%) and temperature (22 ± 2 °C). All experimental protocols were approved by the Local Animal Ethics, Care, and Use Committee for the University of Málaga, Spain (CEUMA 45-2022-A), and conducted in accordance with the EU Directive 2010/63/EU and Spanish Directive (Real Decretory 53/2013).

### Drugs used

The peptides used were freshly prepared in distilled water, which served as the control. Galanin receptor 2 agonist (M1145), NPY1R receptor agonist [Leu31, Pro34] NPY, and GALR2 Antagonist M871 were procured from Tocris Bioscience (Bristol, UK). Peptides were administered once daily for a three-day duration, in line with the methods previously described [46, 47]. Detailed protocols for the intranasal infusion of peptides are provided in the Additional file 1

### Behavioral analysis

#### Assessment of spatial memory in rats

To assess spatial hippocampal memory, we employed the object-in-place task, which is based on spontaneous object exploration behaviors [48]. The advantage of this task over the Morris water maze task is its lesser stress on the rodents, which can interfere with learning and memory performance [49].

Peptides were freshly prepared and administered intranasally three weeks prior to the testing phase (20 μl total volume). Animals were randomly divided into five groups: [1] Control: distilled water; (2) M1145- treated group (132 µg); (3) Y1R agonist-treated group receiving the NPY1R agonist [Leu31- Pro34]NPY (132 µg); (4) M1145 + Y1R: group administered with both substances; (5) M1145 + Y1R + M871: group treated with M1145, [Leu31- Pro34]NPY and the GALR2 antagonist (M871; 132 µg) (n = 6 in each group). Dosage selection was based on prior research [13, 39, 50].

The object-in-place task trials were structured into three phases: habituation, training, and testing [37, 39, 51, 52].

#### Evaluation of hippocampal cell survival

A different cohort of rats was used to examine BrdU-positive cells. Two injections of 5′-Bromo-2′-deoxyuridine (BrdU, cat. no. B5002, Sigma, St. Louis, MO, USA) dissolved at 15 mg/mL in a sterile 0.9% NaCl solution were administered intraperitoneally (i.p.) during the ad libitum feeding period at 50 mg/kg body weight dose (every 2 h over three days, starting at 9:00 AM). The procedures used were based on previously published protocols Three weeks after the after the intranasal infusion, rats were deeply anesthetized with pentobarbital (Mebumal, 100 mg/kg, i.p.) and transcardially perfused with 4% PFA (para-formaldehyde (wt./vol, Sigma Aldrich, St. Louis, MI, USA)). Using a Cryostat (HM550, Microm International, Walldorf, Germany) the brains were coronally sliced (30 μm-thick) through the dorsal hippocampus (posterior in primates) (− 1.60 to − 5.30 Bregma; Paxinos and Watson, 1998). These procedures are based on previously published protocols [22, 45, 53, 54].

Rats were randomly distributed into five experimental groups: (1) Control: distilled water; (2) NPY1R agonist-treated group receiving the NPY1R agonist [Leu^31^- Pro^34^] NPY (132 µg); (3) M1145- treated group (132 µg); (4) Y1R + M1145: group administered with both substances; (5) Y1R + M1145 + M871: group treated with M1145, [Leu^31^- Pro^34^] NPY and the GALR2 antagonist (M871; 132 µg) (n = 4 in each group).

### Immunohistochemistry

Brain sections were incubated free-floating in saline sodium citrate buffer (pH 6; 10 nM sodium citrate) for 90 min at 65 °C, followed by 30 min with 0,6% H2O2 to remove endogenous peroxidases. After 30 min in 2 M hydrochloric acid (HCl) to denature deoxyribonucleic acid (DNA), sections were incubated for neutralization with 0.1 M sodium borate (pH 8). Then, slices were incubated at 4◦C overnight with a primary antibody against BrdU (Abcam, ab152095, 1:1000) in 2,5% donkey serum. Following additional washed with PBS and incubated with a secondary antibody for 90 min (biotinylated anti-rabbit IgG, 1/200, Vector Laboratories), sections were amplified with ExtrAvidin peroxidase (Sigma, St. Louis, MO, USA) diluted 1:100 in darkness at room temperature for one hour. Immunolabeling was exposed with 0.05% diaminobenzidine (DAB; Sigma) and 0.03% H_2_O_2_ in PBS. After various washes, sections were mounted on gelatin-coated slides, dehydrated in graded alcohols, and cover-slipped in DePeX mounting medium (Merck Life Science SLU, Darmstadt, Germany). BrdU-labelled cells with morphological characteristics of glial precursors, i.e. small, irregularly shaped cell bodies, were excluded. Only round, regularly shaped BrdU-positive nuclei located in the DG were counted since new granule cells migrate approximately 2 cell body widths from the SGZ into the granule layer [55]. As previously described, BrdU-labelled cells were analyzed using the optical fractionator method in unbiased stereological microscopy (Olympus BX51 Microscope, Olympus, Denmark) [36, 37, 39] (see Additional file 1).

### Double immunofluorescence

To study the the fate of these newly generated cells we performed double immunolabelling. Procedures for immunofluorescence were previously described [34, 35, 37–39]. Briefly, an initial incubation with blocking (5% goat serum) and permeabilization (0.3% triton X100 in PBS) solutions was performed for 60 min each. Pair of primary antibodies rabbit anti-BrdU (Abcam, ab152095, 1:1000)/mouse anti-DCX (C-18, Santa Cruz, 1:500) or rabbit anti-BrdU (Abcam, ab152095, 1:1000)/mouse anti-NeuN (Abcam, ab1042241, 1:1000) were used to incubate the sections for 24 h, at 4 °C. Then, incubations were performed with proper secondary antibodies: Donkey anti-mouse AlexaFluor 488 (Abcam, ab150105, 1:200) and Donkey anti-rabbit AlexaFluor 647 (Abcam, ab150075, 1:200). Sections were mounted on slides with a fluorescent mounting medium with 4ʹ,6-diamidino-2-phenylindole (DAPI) to detect nuclei (Abcam, ab104139). BRDU/DCX- and BRDU/NeuN double-stained cells in the DG were quantified using z-scan confocal microscopy (Leica Stellaris 8) at 40×magnification. The entire length of the DG was assessed through the septo-temporal axis of the hippocampus, analyzing at least four representative 150 μm, evenly spaced sections per animal. All analyses were conducted in sequential scanning mode to eliminate potential cross-bleeding between channels. Each cell was scrutinized using a multi-channel configuration, and colocalization with NeuN or GFAP was verified by examining multiple optical planes for each cell on the z-axis. Z-stacks were generated at 0.85 μm intervals throughout the 30 μm section to ensure accurate double-labeling of BrdU-IR cells [37, 56].

### Assessment of hippocampal doublecortin (DCX)-labeled newborn neurons

In adult hippocampal neurogenesis, newly generated neuronal cells undergo a phase marked by transient DCX expression, which is evident during the initial stages of neuronal differentiation. This aligns with the understanding that DCX expression in adult hippocampal neurogenesis is confined to the neuronal lineage. Therefore, the assessment of DCX-positive cells can serve as an indicator of the neurogenic effects induced by a specific treatment [57, 58]. The procedure was performed as described for BrdU immunohistochemistry. Briefly, different free-floating sections were incubated for antigenical retrieval at 65 °C during 90 min in saline sodium citrate buffer (pH 6; 10 nM sodium citrate). After this procedure to remove endogenous peroxidases, the slices were treated 30 min in 0.6% H_2_O_2_. Then, a set of slices were incubated at RT overnight with a primary antibody rabbit anti-DCX (Abcam, ab18723, 1:2000) in 2.5% donkey serum. After several washes with PBS, the slices were incubated with a secondary antibody for 90 min (biotinylated anti-rabbit IgG, 1:200, B8895, Sigma, St. Louis, MO, USA). Then, ExtrAvidin peroxidase (1:100, Sigma, St. Louis, MO, USA) was used to amplify the specific signal for one hour at room temperature in darkness. Detection was performed with 0.05% diaminobenzidine (DAB; Sigma) and 0.03% H_2_O_2_ in PBS. After several washes, slices were mounted on gelatin-coated slides, dehydrated in graded alcohols, and cover-slipped with DePeX mounting medium (Merck Life Science SLU, Darmstadt, Germany). DCX-labeled cells were studied using the optical fractionator method in unbiased stereological microscopy (Olympus BX51 Microscope, Olympus, Denmark), as described above.

### Categorization of dendritic morphology of DCX-positive cells

**Fig. 1:**
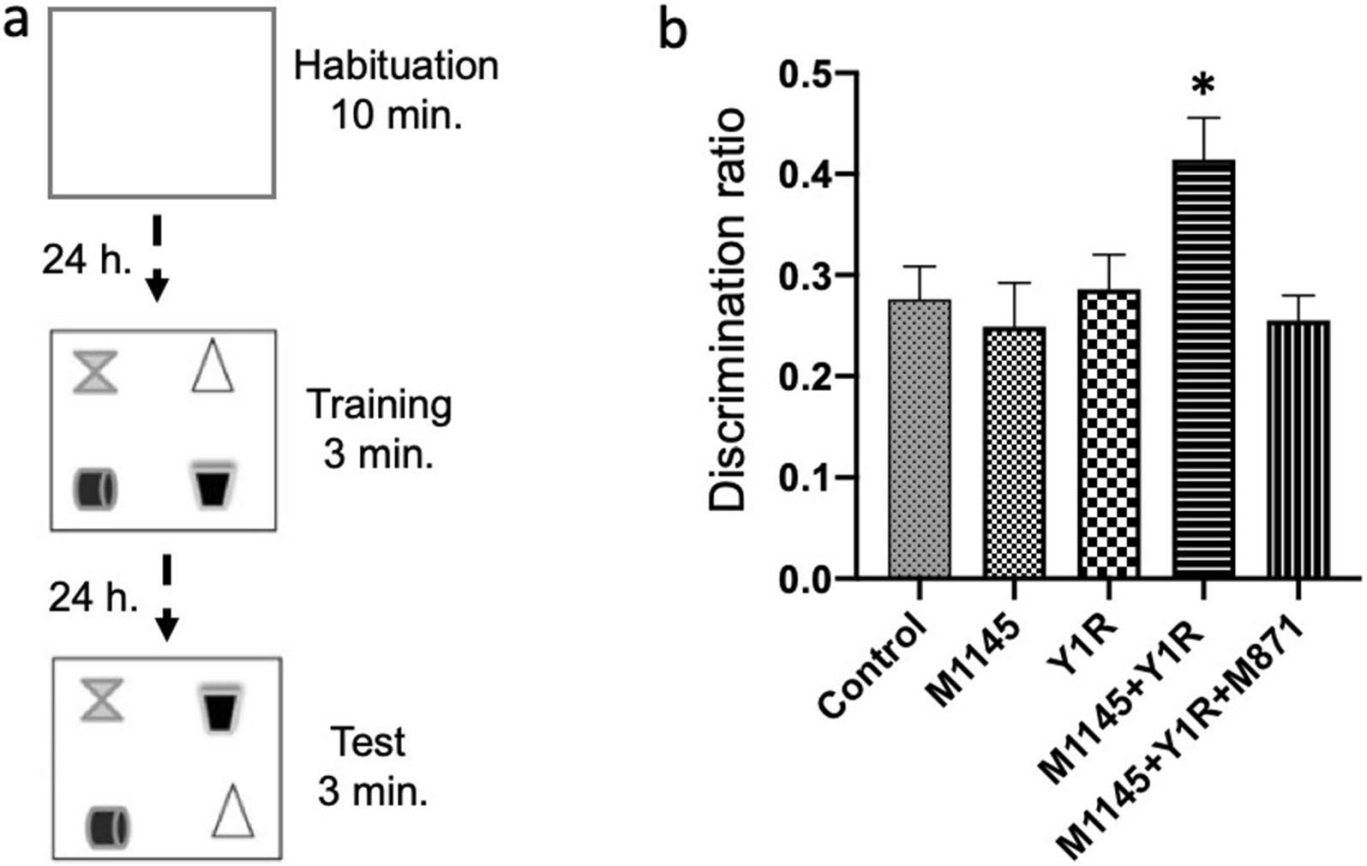
Evaluating Spatial Memory Following Combined Intranasal Administration of NPY1R and GALR2 Agonists in the Object-in-Place Memory Task **a** A schematic representation of the sequential phases in the object-in-place memory task, spaced 24 h apart. The habituation phase allows for free exploration without objects for ten minutes. The training phase then introduces four different objects for a three-minute exploration period. Finally, the test phase involves a three-minute exploration with two of the original objects switched in position. **b** Performance metrics for the object-in-place task, demonstrating the capacity of rats to discern the switched objects after three weeks of intranasal infusion with NPY1R and GALR2 agonists. Notably, the co-administration of M1145 (Galanin 2 receptor agonist, 132 µg) and NPY1R (Y1R receptor agonist [Leu31-Pro34]NPY, 132 µg) resulted in improved performance on the object-in-place task. However, this enhancement was counteracted by the addition of M871 (GALR2 antagonist, 132 µg). Data are expressed as the mean ± SEM of the discrimination ratio during the test phase, based on a sample size of 6 animals per group. *p < 0.05, indicating a significant difference compared to the rest of the groups, as determined by one-way ANOVA and post-hoc Newman–Keuls test. Abbreviations: Control = Distilled water; M1145 = Galanin 2 receptor agonist 132 µg; Y1R = NPY1R receptor agonist [Leu31-Pro34]NPY 132 µg; M1145 + Y1R = Co-administration of M1145 and NPY1R; M1145 + Y1R + M871 = Co-administration of M1145, Y1R and GALR2 antagonist M871, 132 µg.

**Fig. 2:**
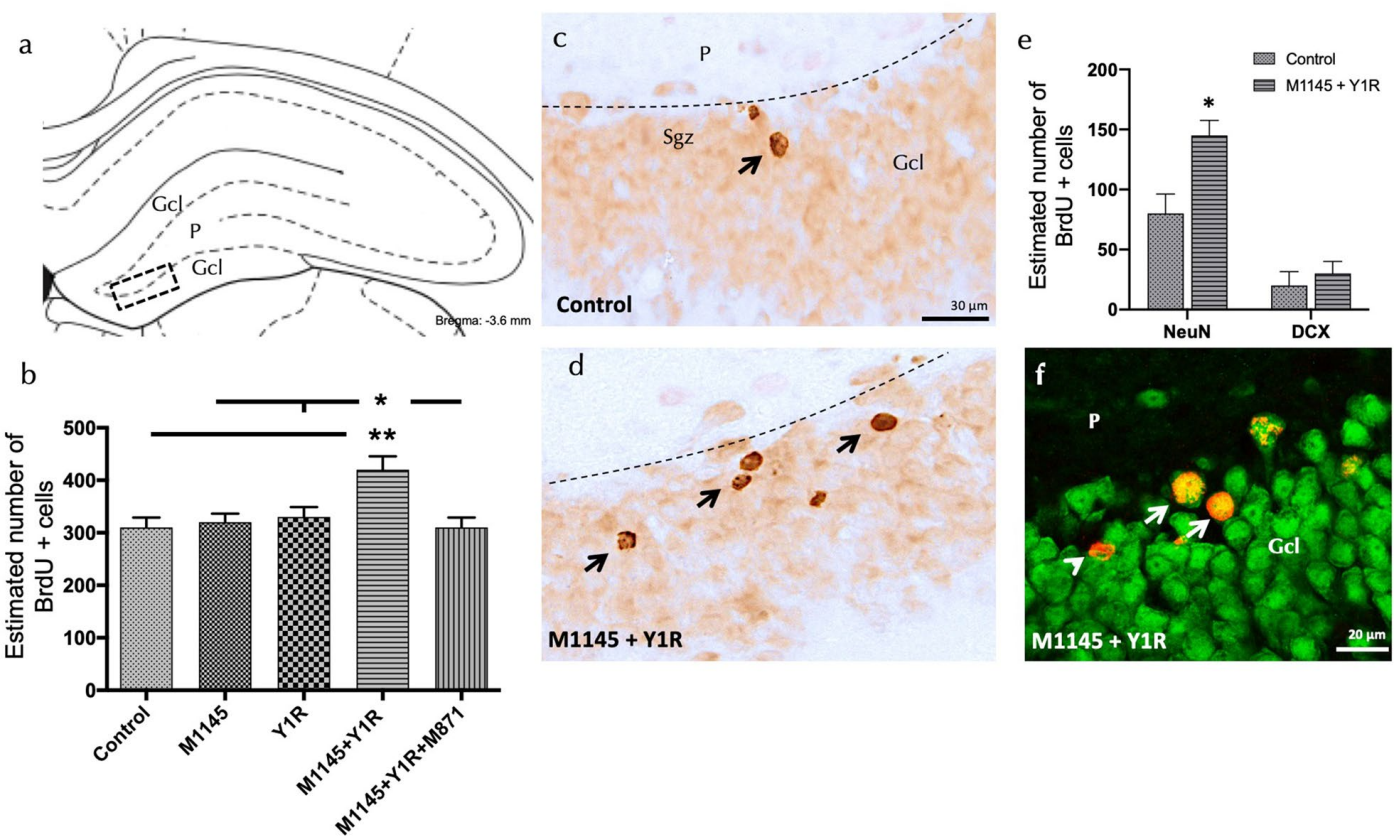
Enhanced Cell Survival in the Dorsal Dentate Gʹyrus Following Intranasal Co-administration of Galanin Receptor 2 and NPY1R Agonists in Adult Rats. 5-Bromo-2ʹ-deoxyuridine (BrdU) immunolabeling following intranasal administration of the Galanin 2 receptor agonist (M1145) and NPY1R receptor agonist, either singularly or in combination, with or without the GAL 2 receptor antagonist (M871). **a**, **d** Most BrdU-positive cells were situated in the granular cell layer (Gcl) of the dentate gyrus in the dorsal hippocampus, exhibiting round and regularly shaped BrdU-positive nuclei (Bregma: − 5.6 mm; as per Paxinos and Watson stereotaxic atlas, 2006). **b** Quantitative assessment of total BrdU-immunoreactive (IR) cells in the dentate gyrus of the dorsal hippocampus, after respective administrations of Control, M1145, NPY1R agonist [Leu31-Pro34]NPY, or co-administration of both agonists with or without M871. Data are presented as mean ± SEM. The co-administration of M1145 and the NPY1R agonist increased the number of BrdU-positive cells in the dorsal hippocampus compared to the effects of the individual peptides and the control group. However, this effect was nullified by the GALR2 antagonist M871. Statistical significance was determined through one-way ANOVA followed by Newman-Keuls post-hoc test, with *P < 0.05 vs M1145, NPY1R and M1145 + Y1R + M871; **P < 0.01 vs Control. **d** M1145 and NPY1R agonist intranasal co-administration notably increased BrdU immunolabeling in the Gcl compared to the control group **c**. Arrows in the figure indicate examples of BrdU positive neurons, while dashed lines delineate the Gcl of the dentate gyrus. **e** Quantification of BrdU-IR cells double-labeled with NeuN or DCX in either control or M1145 + Y1R-administered rats revealed that Y1R-GALR2 specifically induces neuronal maturation. Data are represented as mean ± SEM, with *P < 0.05 vs control according to Student’s unpaired t-test. **f** Representative photomicrograph illustrating BrdU + /NeuN + cells (indicated by white arrows) and BrdU-/NeuN + cells (indicated by white arrowheads) in the M1145 and NPY1R agonist group. Abbreviations: Control = Distilled water; M1145 = Galanin 2 receptor agonist 132 µg; Y1R = NPY1R receptor agonist [Leu31-Pro34]NPY 132 µg; M1145 + Y1R = Co-administration of M1145 and NPY1R; M1145 + Y1R + M871 = Co-administration of M1145, Y1R, and GALR2 antagonist M871 132 µg

We categorized DCX-expressing cells into three categories according to the presence and the shape of apical dendrites (Fig. 1A inset and 1C) and their presumed sequential order (Fig. 2). Category “proliferative stage” were cells without dendrites or very short processes, less than one nucleus-wide (< 10 μm).

In category “Intermediate stage” the process was longer than in B, reached into the granule cell layer, even reaching the molecular layer. For “Postmitotic stage” cells acquire a more mature appearance. One thick dendrite reached into the molecular layer and showed a comparatively sparse branching in the molecular layer; or the dendritic tree showed delicate branching and few major branches either near the soma or within the granule cell layer. To categorize the DCX-positive cells into the three distinct morphological subtypes outlined in our study, for each animal, a sample of 50 DCX-labeled cells was examined. The identified cells were then grouped based on their morphological characteristics, and the results were subsequently presented as the percentage of each subtype within the sampled population [59–62].

### Statistical analysis

Data are presented as mean ± SEM, and sample number (n) is indicated in figure legends. GraphPad PRISM 8.0 (GraphPad Software, La Jolla, CA, USA) was used to analyze all data. One-way analysis of variance (ANOVA) followed by the Newman-Keuls comparison post-test was performed. To analyze the effects of different treatments (Factor A) and the dendrite categories (Factor B) two-way ANOVAs followed by Student—Newman Keuls post-hoc test were made. For comparing two experimental conditions, Student’s unpaired t-test statistical analysis was achieved. Differences were considered significant at p < 0.05 (*p < 0.05 **p < 0.01 ***p < 0.001).

## Results

### Enhanced spatial memory performance following GALR2 and NPY1R agonist intranasal infusion

We administered the object-in-place task three weeks post intranasal (i.n.) infusions. The test involved a habituation phase where rats explored freely for 10 min without objects, a training phase with four different objects, and a test phase where two objects had exchanged positions to assess memory performance (Fig. 1a).

Notably, intranasal infusion of GalR2 agonist M1145 and NPY1R post-acquisition phase significantly improved object-in-place memory consolidation three weeks following treatment compared to other groups (one-way ANOVA, F4, 25 = 3.60, p < 0.05; Newman–Keuls post-hoc test: p < 0.05; Fig. 1b). However, the administration of M1145 or the NPY1R agonist alone demonstrated no effect on the object-in-place memory task (Fig. 1b) compared to the control group.

Moreover, our analysis of total exploration time during training and test sessions showed no significant alterations in the animals' exploration capacity or spontaneous motor behavior following the treatments.

### GALR2 and NPY1R agonist intranasal co-administration promotes survival of mature neurons in the dorsal hippocampus

Our investigation into the cellular mechanisms underpinning these behavioural effects led us to assess the impact of GALR2 and NPY1R agonist intranasal co-administration on adult dorsal hippocampal cell proliferation using the thymidine analogue 5-Bromo-2'-deoxyuridine (BrdU)(Fig. 2a).

Intranasal co-administration of M1145 and the NPY1R agonist significantly increased the number of BrdU-IR profiles in the subgranular zone (Sgz) of the dentate gyrus compared to the control group, as well as the M1145 and NPY1R agonist alone groups (one-way ANOVA, F4, 15 = 5.34, p < 0.01, Newman-Keuls post-hoc test: p < 0.05) (Fig. 2b–d). This increase was completely blocked with the co-treatment of GALR2 antagonist M871, indicating the participation of GALR2 in the M1145/NPYY1R agonist interaction to stimulate cell proliferation.

Additionally, our study into the cellular types affected by the intranasal infusion of M1145 and the NPY1R agonist revealed that the number of BrdU + /NeuN + cells increased significantly after treatment compared to the control group, indicating a preference for newly generated cells to differentiate towards a neuronal lineage (mature neurons).

### Increased doublecortin-positive cells in the dorsal hippocampus Following NPY1R and GALR2 agonist intranasal infusion

**Fig. 3:**
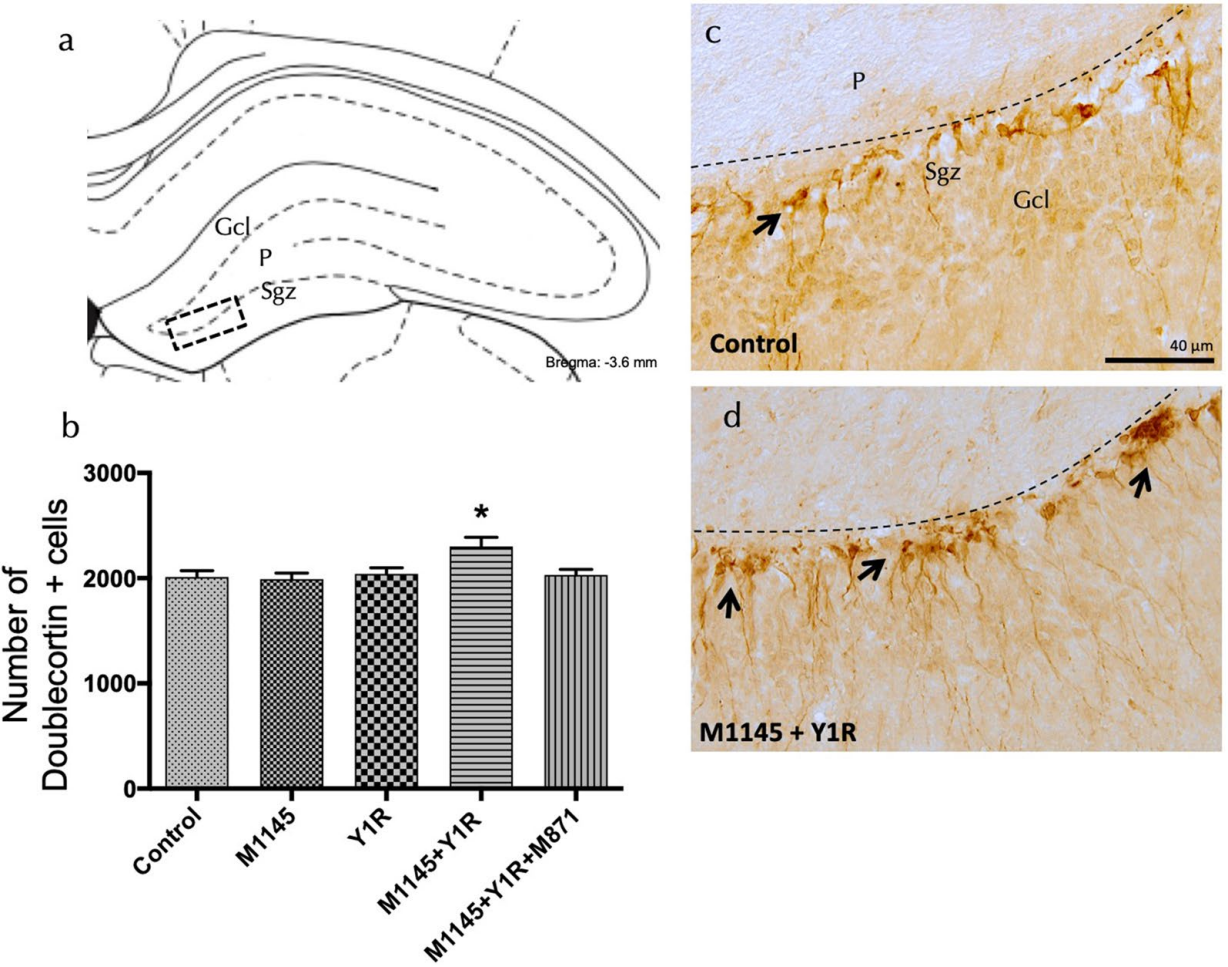
Expression of Doublecortin (DCX) in Dorsal Dentate Gyrus Following Intranasal Co-administration of Galanin Receptor 2 and NPY1R Agonists. This figure presents an examination of DCX-labeled cells in the dorsal dentate gyrus after intranasal administration of the Galanin 2 receptor agonist (M1145) and NPY1R receptor agonist, either alone or in combination, and with or without the GAL 2 receptor antagonist (M871). **a**, **d** DCX-positive cells were found in the subgranular zone (Sgz) of the dentate gyrus, along the junction between the granular cell layer (Gcl) and the polymorphic layer (P) (Bregma: − 5.6 mm; according to Paxinos and Watson stereotaxic atlas, 2006). **b** A quantitative analysis of total DCX-immunoreactive (IR) cells in the dentate gyrus of the dorsal hippocampus was conducted following administrations of Control, M1145, NPY1R agonist [Leu31-Pro34]NPY, or co-administration of both agonists with or without M871. Data are presented as mean ± SEM. Intriguingly, the co-administration of M1145 and the NPY1R agonist resulted in a notable increase in the number of DCX-positive cells in the dorsal hippocampus in contrast to the effects of individual peptides and the control group. This effect, however, was offset by the GALR2 antagonist M871. Statistical significance was calculated via one-way ANOVA followed by Newman-Keuls post-hoc test, with *P < 0.05 vs the rest of the groups **d** The intranasal co-administration of M1145 and NPY1R agonist noticeably increased DCX immunolabeling compared to the control group **c**. Arrows indicate examples of DCX positive neurons, and dashed lines mark the Gcl of the dentate gyrus. Abbreviations: Control = Distilled water; M1145 = Galanin 2 receptor agonist 132 µg; Y1R = NPY1R receptor agonist [Leu31-Pro34]NPY 132 µg; M1145 + Y1R = Co-administration of M1145 and NPY1R; M1145 + Y1R + M871 = Co-administration of M1145, NPY1R, and GALR2 antagonist M871 132 µg.

Our examination into changes in the expression of hippocampal doublecortin (DCX)-labeled newborn neurons in the dorsal hippocampal dentate gyrus (DG) post M1145 and/or NPY1R agonist intranasal administration revealed a significant increase in the total number of DCX-labeled cells post intranasal infusion of M1145 and NPYR1 agonist compared to the rest of the groups (one-way ANOVA, F4, 15 = 3.79, p < 0.05, Newman-Keuls post-hoc test: p < 0.05) (Fig. 3a–d).

### NPY1R and GALR2 agonist intranasal infusion promotes neuronal differentiation of DCX—positive cells in the dorsal hippocampus

**Fig. 4:**
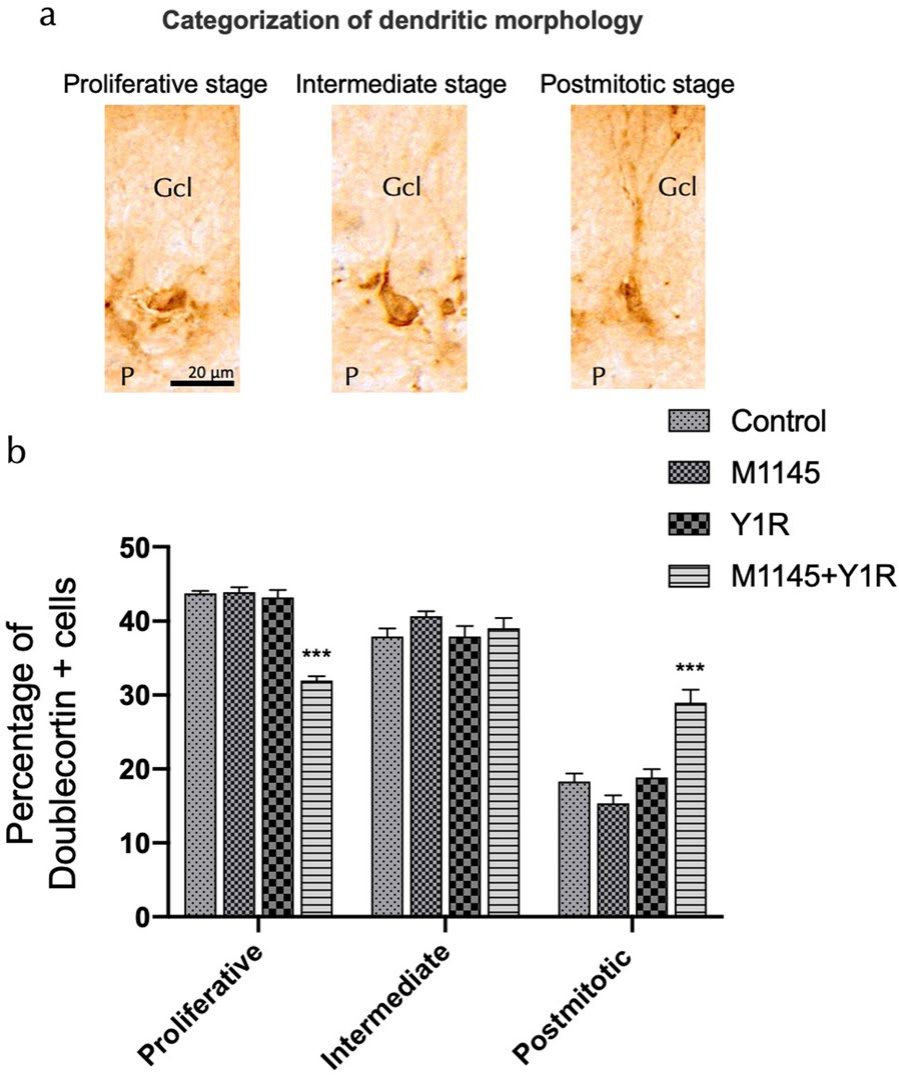
Intranasal Infusion of NPY1R and GALR2 Agonists Facilitates Neuronal Differentiation of DCX-Positive Cells in the Dorsal Hippocampus, as Evidenced by Alterations in Dendritic Morphology. **a** Categorization of DCX-labelled cells was based on their dendritic morphology, specifically delineating between the proliferative, intermediate, and post-mitotic stages of neuronal differentiation. **b** Quantitative analysis revealed a significant reduction in DCX-labelled cells devoid of dendrites or those with dendrites shorter than the soma size in rats treated with a combination of M1145 and NPY1R agonist, compared to other groups. Data are expressed as the percentage of each category. This was statistically significant as determined by a two-way ANOVA (interaction F6,36 = 30.60, p < 0.001; row factor F2,36 = 436.5, p < 0.001, Newman-Keuls post-hoc test: p < 0.001). In contrast, an elevation in the proportion of mature cells was evident in the M1145-NPY1R-treated rats relative to the control group (Newman-Keuls post-hoc test: p < 0.001). Abbreviations: Control = Distilled water; M1145 = Galanin 2 receptor agonist 132 µg; Y1R = NPY1R receptor agonist [Leu31-Pro34]NPY 132 µg; M1145 + Y1R = Co-administration of M1145 and NPY1R; M1145 + Y1R + M871 = Co-administration of M1145, NPY1R, and GALR2 antagonist M871 132 µg

Given the DCX expression, we examined if M1145 and NPY1R agonist would modify dendrite organization in new neurons within the DG. The DCX-labelled cells were categorized based on their dendrite morphology into proliferative, intermediate, and post-mitotic phases of differentiation (Fig. 4a). Quantification revealed a relative decrease in DCX-labelled cells lacking dendrites or cells with dendrites shorter than soma size in M1145-NPY1R-treated rats compared to the rest of the groups (two-way ANOVA, interaction F6,36 = 30.60 p < 0.001; row Factor F2,36 = 436.5 p < 0.001, Newman-Keuls post-hoc test: p < 0.001; Fig. 4b). Conversely, a relative increase was observed in more mature cells from the M1145-NPY1R-treated rats compared to the vehicle group (Newman-Keuls post-hoc test: p < 0.001; Fig. 4b). Notably, in cells with dendrites larger than soma size, the relative proportions were not affected by the GALR2 and/or NPY1R agonists (Fig. 4b).

These findings suggest that M1145 and NPY1R agonist treatment impacts more mature cells, resulting in a decrease in cells without dendrites, which is balanced by an increase in DCX-labelled cells exhibiting more mature dendrite morphology.

## Discussion

Our study provides indications that intranasal co-administration of GALR2 and NPY1R agonists enhances spatial memory and promotes neuronal survival and differentiation in the dentate gyrus of the dorsal hippocampus.

Previously it has only been possible to observe a short-term enhancement in object-in-place memory consolidation after intracerebroventricular agonist treatment with GALR2 and NPY1R [37, 39]. It is of interest that in this work from 2022, the separate icv administration of either the M1145 agonist or the NPY1R agonist did not result in significant memory improvements. It seems possible that with combined treatment allosteric enhancement may develop between GalR2 and NPY1R increasing their signaling which can improve the memory consolidation observed. The results underline a synergistic effect between the postjunctional GalR2 and NPY1R at the trans membrane and cytoplasmatic levels which can be one mechanism for improving consolidation of spatial memory based on intranasal administration of the two neuropeptides. Our findings indicate the importance of reaching the 3 week of neuronal age to achieve hippocampal functionality.

It has been observed that by the third week, there is a considerable population of active neurons that can potentially participate in hippocampal functions in rats [45]. Our cellular analysis in the current study revealed a significant increase in Bromodeoxyuridine (BrdU) immunoreactive profiles in the dentate gyrus, sub-granular zone, after combined treatment of the two neuropeptides using quantitative assessment. It suggests enhanced neurogenesis development. This is a factor closely linked to learning and memory [63].

Furthermore, the majority of these newly generated cells differentiate into mature neurons, as indicated by the rise in proliferating cells determined with BrdU +) and neuronal specific nuclear protein detection (NeuN) after NPY1R-GALR2 injection. This differentiation pattern was supported by the prevalence of NeuN, a marker for mature neurons [64] This was evident at 3 weeks after injection. However, in the current study, the BrdU- doublecortin/DCX) co-expressing cells were relatively low in spite of Doublecortin being a marker for neurons, neural stem cells and neurogenesis [45]. Therefore, the current findings indicate that the memory improvements observed after co-activation of the NPY1R and the GalR2 can mainly be related to an enhanced synergistic signaling of these two receptors. They may be part of a heteroreceptor complex improving memory consolidation leading to long term memory. It can involve also an increased presence and participation of novel mature neurons.

Our decision to employ male rats in this study was based on the understanding of sex differences in adult hippocampal neurogenesis. As delineated by Yagi et al. [62], male rats exhibit a heightened density of BrdU-ir cells at earlier time points (2 h and 1 week) compared to their female counterparts. However, by the 3-week juncture, these disparities diminish, with both males and females showing comparable densities. Furthermore, while the maturation rate of adult-born neurons in the DG (BrdU/NeuN and BrdU/DCX) is accentuated in males at 2 weeks, it equalizes between the genders by the third week [62]. Given our investigative focus on the 3 week mark for neuronal survival, the selection of male rats was strategic, ensuring a more controlled evaluation without the potential confounds of sex-related disparities observed at earlier stages of neurogenesis. Future studies might benefit from a broader inclusion of both sexes to elucidate any clear differences or interactions that might emerge across different experimental conditions or timeframes.

Furthermore, our study highlighted the dendritic morphological changes in DCX-labeled cells post-treatment, implying enhanced functional integration of these neurons into existing circuits. The role of DCX in neuritic growth cone genesis and synapse development [65] suggests that dendritic complexity and length, crucial for neuronal functionality [66] might be modulated by DCX activity. This perspective requires further research to unravel the intricate interplay between cellular survival, maturation, and neuronal integration.

We acknowledge certain limitations in our current study. While our findings based on immunocytochemistry provide valuable insights into the effects of GALR2 and NPY1R agonists on hippocampal neurogenesis, more advanced techniques could offer a deeper understanding of the underlying mechanisms. Techniques such as transcriptomic analysis could help elucidate the molecular pathways activated by these agonists. Additionally, electrophysiological studies might shed light on the functional integration of the newly generated neurons into existing neural circuits. In vivo imaging techniques, such as two-photon microscopy, could provide real-time insights into the dynamics of neuronal development and integration post-agonist administration. We recognize the potential of these advanced methodologies to build a more comprehensive understanding of the role of neuropeptide receptor agonists in neurogenesis and cognitive functions.

In conclusion, our research introduces a promising avenue for memory enhancement and neuronal maturation via the co-administration of GalR2 and NPY1R agonists. Further investigations are essential to elucidate the molecular underpinnings of this synergistic effect and its potential therapeutic applications in learning and memory and in understanding its molecular architecture.

## Conclusions

This study provides evidence that co-administration of GALR2 and NPY1R agonists can enhance spatial memory and promote neuronal survival and differentiation in the dorsal hippocampus of adult rats. Our findings emphasize a synergistic effect between GalR2 and NPY1R receptors in boosting spatial memory consolidation. This synergism aligns with the growing body of research highlighting the potential of intranasal drug delivery as a promising avenue for addressing cognitive deficits and reduced neurogenesis conditions.

Our cellular analyses further reveal that the combined administration of these agonists not only boosts cell survival but also favors the differentiation of newly generated cells into mature neurons. This is evidenced by the increased presence of BrdU + /NeuN + cells and the elevated expression of doublecortin (DCX), a recognized marker for newborn neurons. Additionally, the observed changes in dendritic morphology suggest a potential for enhanced functional integration of these new neurons into existing neural networks.

The data from this study support the potential therapeutic utility of modulating GALR2 and NPY1R receptors in addressing cognitive decline associated with aging. It will be crucial for future research to explore deeper into the specific molecular pathways that drive this synergistic effect, especially in the context of diseases characterized by memory impairment and reduced neurogenesis. Our study serves as an initial step in this direction, presenting a novel strategy for enhancing memory and promoting neuronal health.

### Supplementary Information

**Additional file 1.** Extended methodology.

## Data Availability

The data that support the findings of this study are openly available in the Institutional repository of the University of Malaga (RIUMA) and from the corresponding author upon reasonable request.

## Abbreviations

GALR2: Galanin receptor 2
NPY1R: Neuropeptide Y1 receptor
BrdU: 5-Bromo-2′-deoxyuridine
DCX: Doublecortin
DG: Dentate gyrus
NeuN: Neuronal nuclei (also known as Fox-3)
IEG: Immediate early gene
LTP: Long-term potentiation
