# Supplementary material for: Enhancement of neurogenesis and cognition through intranasal co-delivery of galanin receptor 2 (GALR2) and neuropeptide Y receptor 1 (NPY1R) agonists: a potential pharmacological strategy for cognitive dysfunctions
Source: Behav Brain Funct. 2024 Mar 28;20:6. doi: 10.1186/s12993-024-00230-5 (PMC10976774; doi:10.1186/s12993-024-00230-5)
Supplement: Supplementary file 1 — Additional file 1. Extended methodology. [file 12993_2024_230_MOESM1_ESM.docx]

***Additional file Material***

**Enhancement of Neurogenesis and Cognition through Intranasal Co-Delivery of GALR2 and NPY1R Agonists: A Potential Pharmacological Strategy for Cognitive Dysfunctions**

Raquel Sanchez-Varo1,2,3**;** Jose Erik Alvarez-Contino 1,5; Rasiel Beltran-casanueva 4,5 **;**Estela Díaz-Sánchez1,6 **;** Alexander López-Salas 1,5**;** Miguel Angel Barbancho-Fernández 1,2**;** Pedro Serrano-Castro 2,6,7**;** Kjell Fuxe 4**;** Dasiel Borroto-Escuela 1,2,4,5**;** Natalia García-Casares 1,2,8**;** Manuel Narváez 1,2,6.

1 NeuronLab. Departamento Fisiología Humana, Histología Humana, Anatomía Patológica y Educación Física y Deportiva. Facultad de Medicina, Universidad de Malaga29071, Malaga, Spain

2 Instituto de Investigación Biomédica de Málaga-IBIMA-Plataforma Bionand Universidad de Malaga 29071 Malaga Spain

3 Centro de Investigación Biomédica en Red Sobre Enfermedades Neurodegenerativas (CIBERNED) 28031, Madrid, Spain.

4 Department of Neuroscience, Karolinska Institutet, Stockholm, Sweden.

5 Receptomics and Brain disorders lab, Department of Human Physiology, Sport and Exercise, Faculty of Medicine, University of Malaga, Edificio Lopez-Penalver, Jimenez Fraud 10, 29071 Málaga, Spain.

6 Vithas Málaga., Vithas Málaga. Grupo Hospitalario Vithas, Málaga, Spain,

7 Instituto de Investigación Biomédica de Málaga, Unit of Neurology, Hospital Regional Universitario de Málaga, Málaga, Spain

8 Departamento de Medicina y Dermatología. Facultad de Medicina. Universidad de Málaga. MálagaSpain

Correspondence: mnarvaez@uma.es (M.N.)

**Intranasal administration of peptides**

Galanin receptor 2 agonist (M1145), Y1R receptor agonist [Leu^31^, Pro^34^]NPY, GALR2 Antagonist M871 (Tocris Bioscience, Bristol, UK) were freshly dissolved in 20 μl distilled water. Each rat received 10 μl of them into each nostril with pipetteman and disposable plastic tip (1 mm in diameter) inserted no deeper than 1–1.5 mm into the nostril under light isoflurane anesthesia. Following the infusion, the head of the animal was held in a tilted back position for approximately 15 s to prevent loss of solution from the nares.

**Counting Procedure**

BrdU and DCX-labeled cells were counted with an Olympus BX51 microscope, Olympus, Denmark interfaced with a computer and a colour JVC digital video camera. For stereological analysis, sampling of positive cells was performed throughout the dentate gyrus of the dorsal hippocampus in the rostrocaudal dimension using the optical fractionator, according to Paxinos & Watson atlas coordinates (Paxinos & Watson, 2006). This method combines the optical dissector with a fractionator sampling scheme to exclude volume divergences (Gundersen et al., 1988). Counterstaining with phase contrast allowed delineation of different areas in each section (Paxinos and Watson, 2006). Numbers of positive cells were quantified in at least six representative 150 μm, evenly spaced sections per animal (4 rats per group). A random set of sampling frames with a known area (α frame) was generated for each section using the C.A.S.T. Grid (Olympus; Albertslund, Denmark). After the objects were counted (ΣQ-) the total number of positive cells were estimated as: N = ΣQ- x fs x fa x fh (Gundersen et al., 1988), where fs is the numerical fraction of the section used, fa is the areal fraction and fh is the linear fraction of section thickness. The coefficient of error (CE) for each estimation and animal ranged from 0.05 to 0.1. The total CE of each group ranged from 0.07 to 0.08. Counting of labelled cells was set starting at 5 μm below the surface and focusing through the 20 μm section optical plane, and the number of counting frames used was 90-110 per animal. We have used this stereological procedure is previous studies (Narvaez et al., 2016;Narvaez et al., 2018; Borroto-escuela et al., 2022; Mirchandani-duque et al., 2022).

**References**

Borroto-Escuela, D. O., Fores, R., Pita, M., Barbancho, M. A., Zamorano-Gonzalez, P., Casares, N. G., . . . Narvaez, M. (2022). Intranasal Delivery of Galanin 2 and Neuropeptide Y1 Agonists Enhanced Spatial Memory Performance and Neuronal Precursor Cells Proliferation in the Dorsal Hippocampus in Rats. Front Pharmacol, 13, 820210. doi:10.3389/fphar.2022.820210

Gundersen, H.J., Bagger, P., Bendtsen, T.F., Evans, S.M., Korbo, L., Marcussen, N., Moller, A., Nielsen, K., Nyengaard, J.R., Pakkenberg, B., and Et Al. (1988). The new stereological tools: disector, fractionator, nucleator and point sampled intercepts and their use in pathological research and diagnosis. APMIS 96, 857-881.

Mirchandani-Duque, M., Barbancho, M. A., Lopez-Salas, A., Alvarez-Contino, J. E., Garcia-Casares, N., Fuxe, K., . . . Narvaez, M. (2022). Galanin and Neuropeptide Y Interaction Enhances Proliferation of Granule Precursor Cells and Expression of Neuroprotective Factors in the Rat Hippocampus with Consequent Augmented Spatial Memory. Biomedicines, 10(6). doi:10.3390/biomedicines10061297

Narvaez, M., et al. (2015). "Galanin receptor 2-neuropeptide Y Y1 receptor interactions in the amygdala lead to increased anxiolytic actions." Brain Struct Funct 220(4): 2289-2301.

Narvaez, M., et al. (2016). "Galanin receptor 2-neuropeptide Y Y1 receptor interactions in the dentate gyrus are related with antidepressant-like effects." Brain Struct Funct 221(8): 4129-4139

Narvaez, M., et al. (2018). "A Novel Integrative Mechanism in Anxiolytic Behavior Induced by Galanin 2/Neuropeptide Y Y1 Receptor Interactions on Medial Paracapsular Intercalated Amygdala in Rats." Front Cell Neurosci 12: 119

Paxinos, G., and Watson, C. (2006). *The rat brain in stereotaxic coordinates: hard cover edition.* Elsevier.

Serova, L.; Mulhall, H.; Sabban, E. NPY1 Receptor Agonist Modulates Development of Depressive-Like Behavior and Gene Expression in Hypothalamus in SPS Rodent PTSD Model. *Front Neurosci* **2017**, *11*, 203, doi:10.3389/fnins.2017.00203.

Serova, L.I.; Hansson, E.; Sabban, E.L. Effect of intranasal administration of neuropeptide Y and single prolonged stress on food consumption and body weight in male rats. *Neuropeptides* **2020**, *82*, 102060, doi:10.1016/j.npep.2020.102060.
